# Cancer Cell Expression of Autotaxin Controls Bone Metastasis Formation in Mouse through Lysophosphatidic Acid-Dependent Activation of Osteoclasts

**DOI:** 10.1371/journal.pone.0009741

**Published:** 2010-03-17

**Authors:** Marion David, Estelle Wannecq, Françoise Descotes, Silvia Jansen, Blandine Deux, Johnny Ribeiro, Claire-Marie Serre, Sandra Grès, Nathalie Bendriss-Vermare, Mathieu Bollen, Simone Saez, Junken Aoki, Jean-Sébastien Saulnier-Blache, Philippe Clézardin, Olivier Peyruchaud

**Affiliations:** 1 INSERM, U664, Lyon, France; 2 Université Claude Bernard Lyon 1, Villeurbanne, France; 3 Faculté de Médecine Laennec, Lyon, France; 4 INSERM, U858, Toulouse, France; 5 Centre Hospitalier Lyon Sud, Hospices Civils de Lyon, Pierre Bénite, France; 6 Laboratory of Biosignaling and Therapeutics, Department of Molecular Cell Biology, University of Leuven, Leuven, Belgium; 7 INSERM, U590, Lyon, France; 8 Centre Leon Bérard, Lyon, France; 9 Tohoku University, Sendai, Japan; Leiden/Amsterdam Center for Drug Research, Leiden University, The Netherlands

## Abstract

**Background:**

Bone metastases are highly frequent complications of breast cancers. Current bone metastasis treatments using powerful anti-resorbtive agents are only palliative indicating that factors independent of bone resorption control bone metastasis progression. Autotaxin (ATX/NPP2) is a secreted protein with both oncogenic and pro-metastatic properties. Through its lysosphospholipase D (lysoPLD) activity, ATX controls the level of lysophosphatidic acid (LPA) in the blood. Platelet-derived LPA promotes the progression of osteolytic bone metastases of breast cancer cells. We asked whether ATX was involved in the bone metastasis process. We characterized the role of ATX in osteolytic bone metastasis formation by using genetically modified breast cancer cells exploited on different osteolytic bone metastasis mouse models.

**Methodology/Principal Findings:**

Intravenous injection of human breast cancer MDA-B02 cells with forced expression of ATX (MDA-B02/ATX) to inmmunodeficiency BALB/C *nude* mice enhanced osteolytic bone metastasis formation, as judged by increased bone loss, tumor burden, and a higher number of active osteoclasts at the metastatic site. Mouse breast cancer 4T1 cells induced the formation of osteolytic bone metastases after intracardiac injection in immunocompetent BALB/C mice. These cells expressed active ATX and silencing ATX expression inhibited the extent of osteolytic bone lesions and decreased the number of active osteoclasts at the bone metastatic site. *In vitro*, osteoclast differentiation was enhanced in presence of MDA-B02/ATX cell conditioned media or recombinant autotaxin that was blocked by the autotaxin inhibitor vpc8a202. *In vitro*, addition of LPA to active charcoal-treated serum restored the capacity of the serum to support RANK-L/MCSF-induced osteoclastogenesis.

**Conclusion/Significance:**

Expression of autotaxin by cancer cells controls osteolytic bone metastasis formation. This work demonstrates a new role for LPA as a factor that stimulates directly cancer growth and metastasis, and osteoclast differentiation. Therefore, targeting the autotaxin/LPA track emerges as a potential new therapeutic approach to improve the outcome of patients with bone metastases.

## Introduction

Bone is a common metastatic site for many cancers [1]. Bone metastases are associated with hypocalcaemia due to bone destruction, intractable bone pain and pathological fractures. Tumor cells present at the bone metastatic site stimulate osteoclast-mediated bone resorption, and bone-derived growth factors released from resorbed bone promote tumor growth, leading to the development of a vicious cycle. [2]. Unfortunately, current treatments using powerful anti-resorptive agents (bisphosphonates) fail to provide life-prolonging benefit to patients with bone metastases raising the need for a better knowledge of the molecular mechanisms involved in this pathology [3]. We have recently found that lysophosphatidic acid (LPA) derived from blood platelets acts locally on tumor cells to promote the progression of bone metastases [4]. Among the cell surface specific receptors for LPA (LPA_1–6_) expressed by tumor cells, we demonstrated that LPA_1_ activity prevailed during bone metastasis progression and that targeting this receptor was a new therapeutic strategy against bone metastases [5]. However, how LPA is generated at the tumor site and how LPA produced by tumor cells themselves might contribute to the progression of bone metastases are still unknown.

Autotaxin (ATX, NPP2) belongs to the nucleotide pyrophosphate phosphodiesterase (NPP) family [6]. In addition to the conserved phosphodiesterase (PDE) activity among all NPPs, autotaxin has a unique lysophospholipase D (lysoPLD) activity, allowing the generation of LPA from lysophosphatidylcholine (LPC) [7]. The biological significance of PDE and lysoPLD activities in autotaxin functions remains to be determined. However, the functional relevance of the catalytic activity of autotaxin *in vivo* was recently demonstrated from knockout mice studies showing that autotaxin is responsible for the levels of LPA in the blood circulation [8], [9]. A link between increased lysoPLD activity and the formation of LPA was found in various pathologies such as rheumatoid arthritis [10], neuropathic pain [11], chronic hepatitis C [12] and adipocyte insulin-resistance in obesity [13]. Autotaxin is a glycoprotein initially identified as an autocrine motility factor secreted by human melanoma cells [14], [15]. Increased expression of autotaxin was shown to correlate with increased invasiveness of breast cancer cells [16] and was found to enhance the metastatic potential of ras-transformed 3T3 fibroblasts [17]. Expression of autotaxin mRNA was detected at a basal level in almost all human tissues [18]. Intriguingly, upregulation of autotaxin gene was reported in a large variety of cancers such as glioblastoma [19], aggressive neuroblastoma [20], non small cell lung cancer [21], uveal melanoma associated with poor prognosis [22], thyroid carcinoma [23], hepatocellular carcinoma with metastases [24], and breast cancer [16]. MMTV-*atx* transgenic mice with specifically increased expression of autotaxin in the mammary gland showed an increased in the incidence of spontaneous mammary tumors over a two-year period, illustrating the pro-oncogenic function of autotaxin [25].

Here, we provide experimental evidence that breast cancer cells expressing autotaxin have a selective advantage to induce the formation of osteolytic bone metastases as a result of a novel pro-osteoclastic function of autotaxin-derived product LPA. These results illustrate the role of autotaxin in advanced breast cancers and suggest that targeting the autotaxin/LPA track might provide additional benefit for patients suffering from bone metastases.

## Results

### *De novo* autotaxin expression increases proliferation and invasion of human MDA-B02 breast cancer cells *in vitro*

We have shown previously that MDA-B02 cells do not express autotaxin at steady state [4]. To assess whether or not autotaxin plays a role in the metastasis dissemination of breast tumor cells, we introduced the cDNA of rat autotaxin into MDA-B02 cells. We used the tet-Off-regulated expression system in which autotaxin expression along with the luciferase is achieved specifically in the absence of the repressor, doxycycline [4]. As a unique member of the NPP family, autotaxin exhibits both lysoPLD and phosphodiesterase activities [26], [27]. Therefore, as a control cell line we transfected MDA-B02 cells with an expression vector coding for mouse NPP1, which acts as a nucleotide phosphodiesterase but that lacks for the lysoPLD activity.

**Figure 1:**
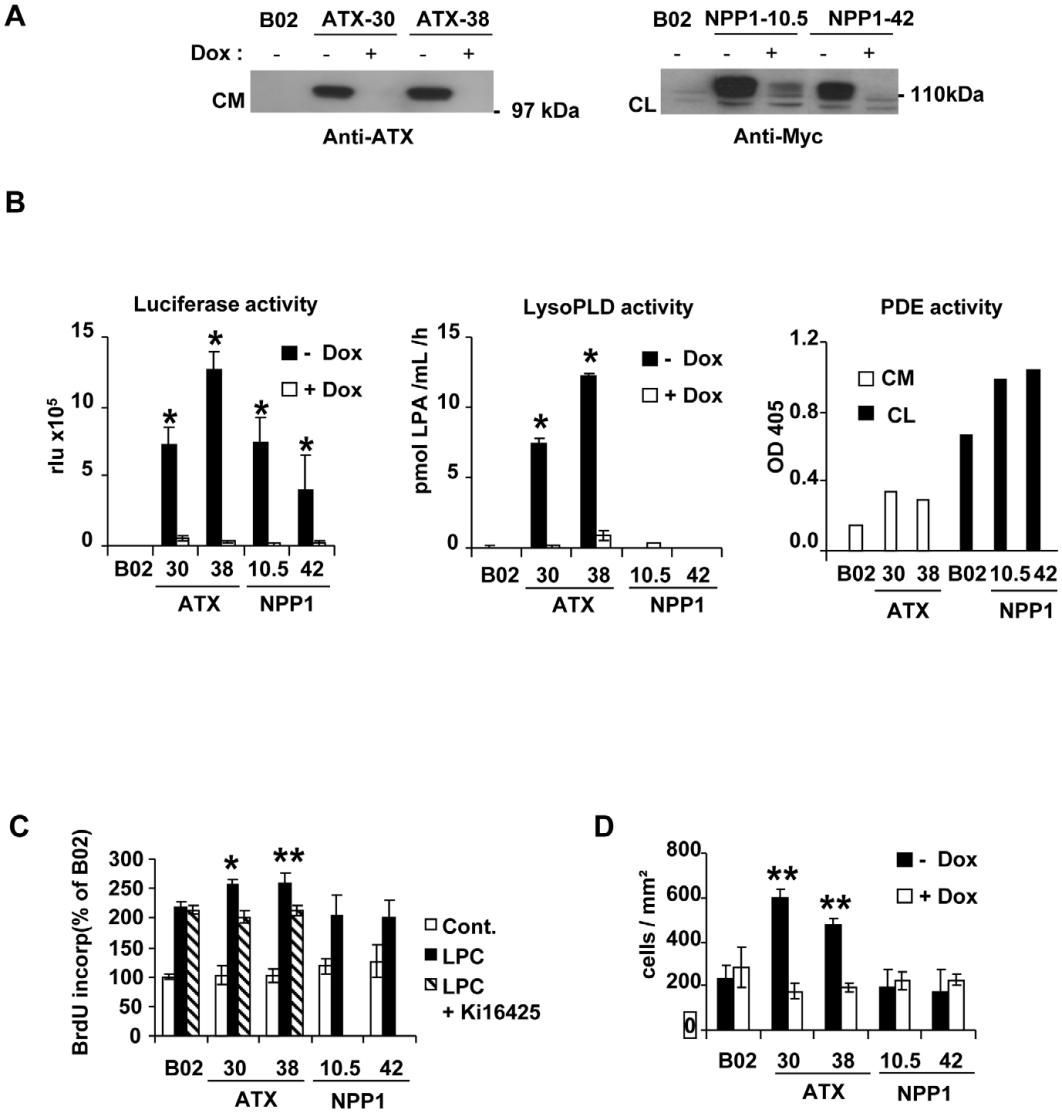
Characterization of forced expression of autotaxin in human breast cancer MDA-B02 cells. (A) Cells transfected with bidirectional expression vectors pBiL-ATX or pBil-NPP1 were plated with (+) or without (-) doxycycline (Dox). Proteins from conditioned media (CM) or lysates of tumor cells (CL) of two stable clones (no. 30 and no. 38 to ATX, no. 10.5 and no. 42 to NPP1) were electrophorezed then immunoblotted with an anti-ATX antibody (Left panel) or anti-Myc antibody (Right panel). (B) Quantifications of luciferase activity (Left panel), lysoPLD activity (Middle panel) and PDE activity (Right panel) in each clone and parental MDA-B02 cells. (C) Cell proliferation was stimulated with LPC (10 µM) in absence or presence Ki16425 (10 µM). Results are expressed as the % of BrdU incorporation compared to unstimulated MDA-B02 parental cells. Data correspond to the mean ± SD of 6 replicates and are representative of at least 3 independent experiments. (D) Cell invasion was stimulated with 10% FBS used as chemoattractant. Results are the mean ± SD of cells of 3 replicates and are representative of at least 3 independent experiments. Data are expressed as the number of cells/mm^2^. *, *P*<0.05. **, *P*<0.01

For each construct, we selected two stable clones: MDA-B02-ATX clone no. 30 and no. 38, and MDA-B02-NPP1 clones no. 10.5 and no. 42. The expression of autotaxin as a secreted protein and NPP1 as a membrane-bound protein were confirmed by western blotting using specific antibodies (Figure 1A). Clone selections were based on high luciferase expression in absence of doxycycline (Figure 1B). As expected, we observed that in the absence of doxycycline only MDA-B02-ATX clones acquired a lysoPLD activity (Figure 1B), whereas both MDA-B02-ATX and MDA-B02-NPP1 clones had an increased PDE activity, as compared to parental cells (Figure 1B).

We have shown previously that the treatment with LPA increases the proliferation of MDA-B02 cells [4]. Here, we observed that, in the absence of doxycycline, LPC had a mitogenic activity by itself on both the parental MDA-B02 cells and transfected clones (Figure 1C). This findings might reflect the expression LPC receptors (GPR4, TDAG8) in MDA-B02 cells as well as in breast tumors [28]. However, LPC-dependent proliferation was further increased in MDA-B02-ATX cells, but not in MDA-B02-NPP1 cells or parental cells. Moreover, the treatment of cells with Ki16425, an antagonist of LPA_1_ and LPA_3_ receptors, totally blocked autotaxin-dependent but not LPC-dependent cell proliferation (Figure 1C). This indicated that recombinant ATX expressed by MDA-B02-ATX cells produced active LPA that stimulated cell proliferation through a LPA_1–3_ receptor-dependent pathway.

Autotaxin has been implicated in the invasiveness of ras-transformed fibroblasts and breast cancer cells [16], [17]. We observed that, in absence of doxycycline, MDA-B02-ATX clones had a significantly higher capacity to invade Matrigel™ in response to FBS than in the presence of doxycycline (Figure 1D). The gain of invasiveness of MDA-B02-ATX clones was also observed when compared to parental cells and MDA-B02-NPP1 clones, placed either in absence or presence of doxycycline. These results confirmed that autotaxin increased the invasive potential of breast cancer cells by a mechanism that required its lysoPLD activity.

### *De novo* autotaxin expression enhances *in vivo* MDA-B02 bone metastasis formation

**Figure 2:**
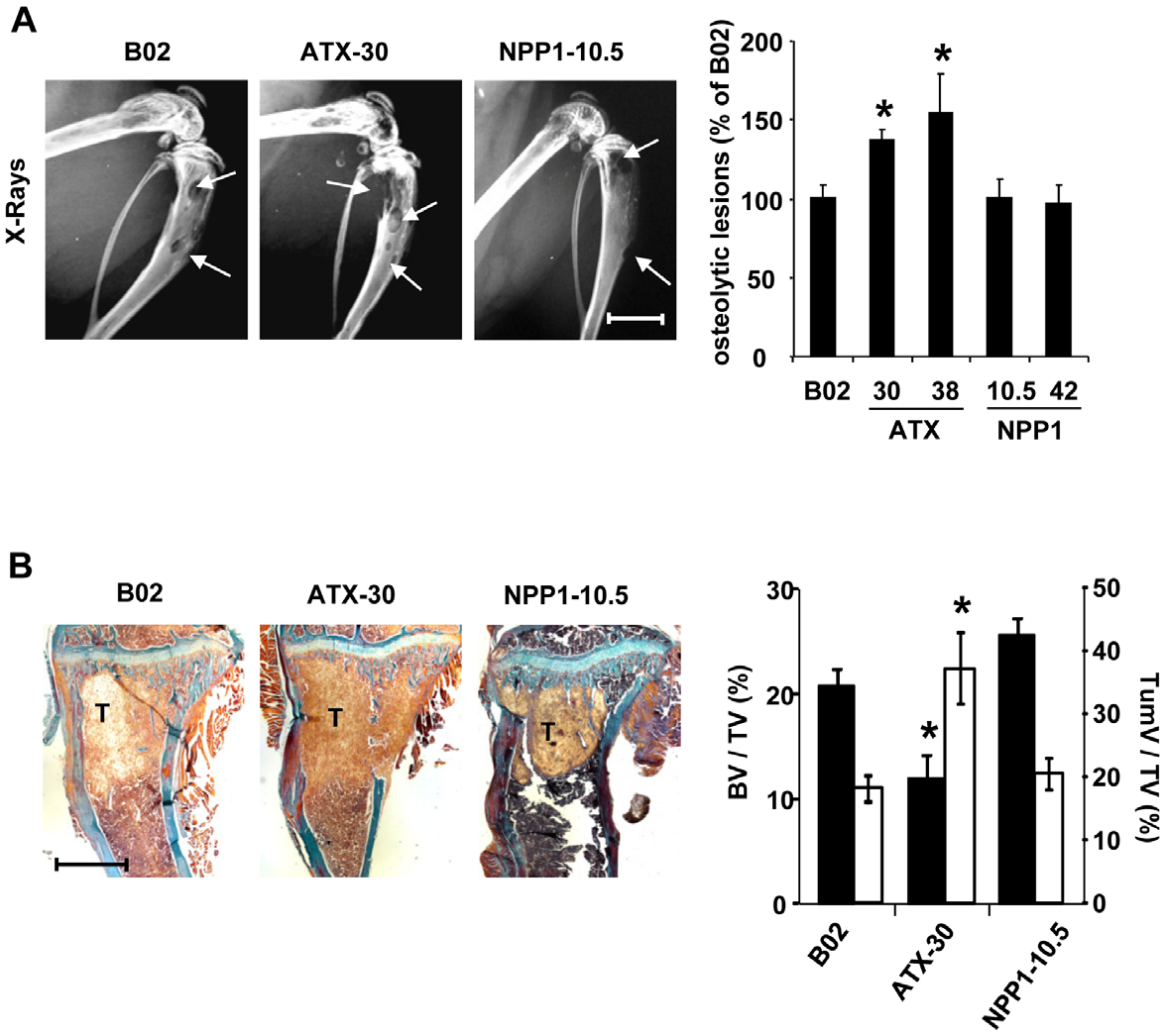
Effect of forced expression of autotaxin on osteolytic bone metastasis formation of MDA-B02 cells. (A) (Left panels) Representative radiographs of hind limbs from metastatic mice bearing MDA-B02 cells or MDA-B02-ATX clone no. 30 or MDA-B02-NPP1 clone no. 42, 29 days after tumor cell inoculation. Osteolytic lesions are indicated by arrows (scale bar: 0.5 cm). (Right panel) Quantification of osteolytic lesion areas on radiographs in metastatic animals. Data correspond to the mean ± SE of two independent experiments of 7 to 10 animals per group. (B) (Left panels) Representative bone histology of Goldner's trichrome-stained tibial metaphysis from metastatic animals. Bone is stained in blue; bone marrow and tumor cells are stained in red. (scale bar: 1 mm). (Right panel) Histomorphometric analysis of metastatic hindlimbs using the bone volume/tissue volume ration (BV/TV, black bars and left axis) and the tumor volume/tissue volume ratio (TumV/TV, open bars and right axis) as referents. Values are the mean ± SE of 7–10 animals per group representative of two independent experiments. *, *P*<0.05.

**Figure 3:**
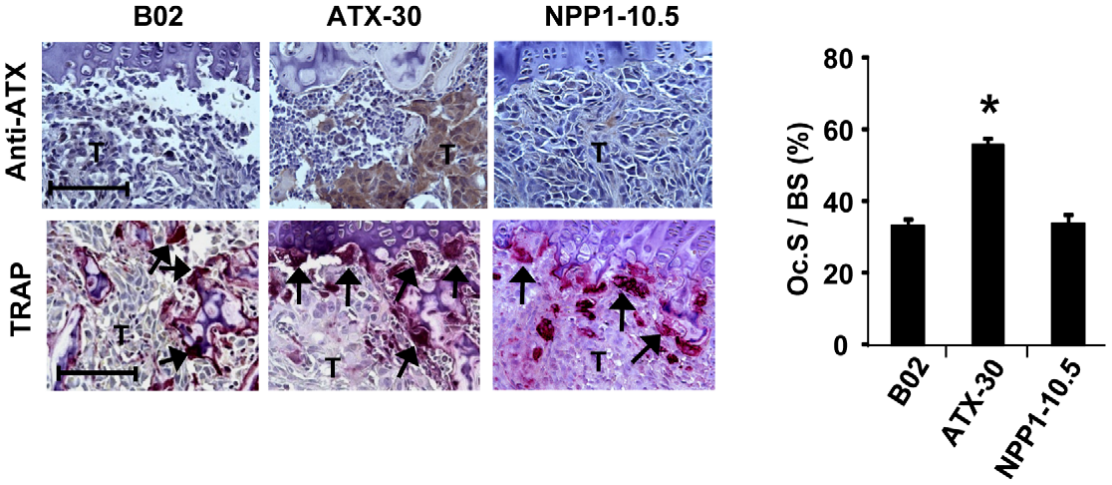
Effect of forced expression of autotaxin *in vivo* on MDA-B02 cells increased the formation osteoclasts at the bone metastatic site. (Upper left panels) Representative immunohistological examination of proximal tibia sections from metastatic animals 29 days after tumor cell inoculation, using the anti-ATX antibody 4F1. T indicates tumor cells. (Lower left panels) Representative histological examination of TRAP-stained proximal tibia sections from metastatic animals. T indicates tumor cells. Bone is stained in dark blue and osteoclats are stained in red (arrows). (Right panel) Quantification of active-osteoclast resorption surface per trabecular bone surface (Oc.S/BS). Results are the mean ± SE of 8–9 animals per group. *: *P*<0.05. Scale bars: 200 µm.

We have previously demonstrated that LPA derived from platelets supports the progression of bone metastases mediated by MDA-B02 cells in mice [4]. We hypothesized that elevated tumor cell-derived lysoPLD activity might also promote bone metastasis. Thirty two days after the intravenous inoculation of tumor cells into *nude* mice, radiographic analyses revealed that animals bearing MDA-B02-ATX clones exhibited a 40% to 70% increase in the extent of osteolytic lesions, as compared to that seen with MDA-B02-NPP1 clones and parental cells (Figure 2A). Histological examinations and histomorphometric analyses confirmed the radiographic observations and showed that *de novo* expression of autotaxin by breast cancer cells resulted in a reduction of bone volume (BV/TV) and increased skeletal tumor burden (Figure 2A). We observed no difference on legs of metastatic animals bearing MDA-B02-NPP1 clones compared to MDA-B02 parental cells at the histological level (Figure 2B). We have previously shown that LPA stimulates the potency of tumor cells to increase the recruitment of osteoclasts at the bone metastatic site [4]. Here, we observed that the surface of active osteoclasts per trabecular bone area located at the bone/tumor cell interface was increased in animals bearing MDA-B02-ATX clones, as compared to that observed in mice bearing parental or NPP1-expressing tumor cells (Figure 3).

Altogether, our results indicated that increased expression of autotaxin by MDA-B02 cells enhanced the formation of osteolytic bone metastases.

### Downregulation of autotaxin expression inhibits invasion but not proliferation of mouse 4T1 breast cancer cells

**Figure 4:**
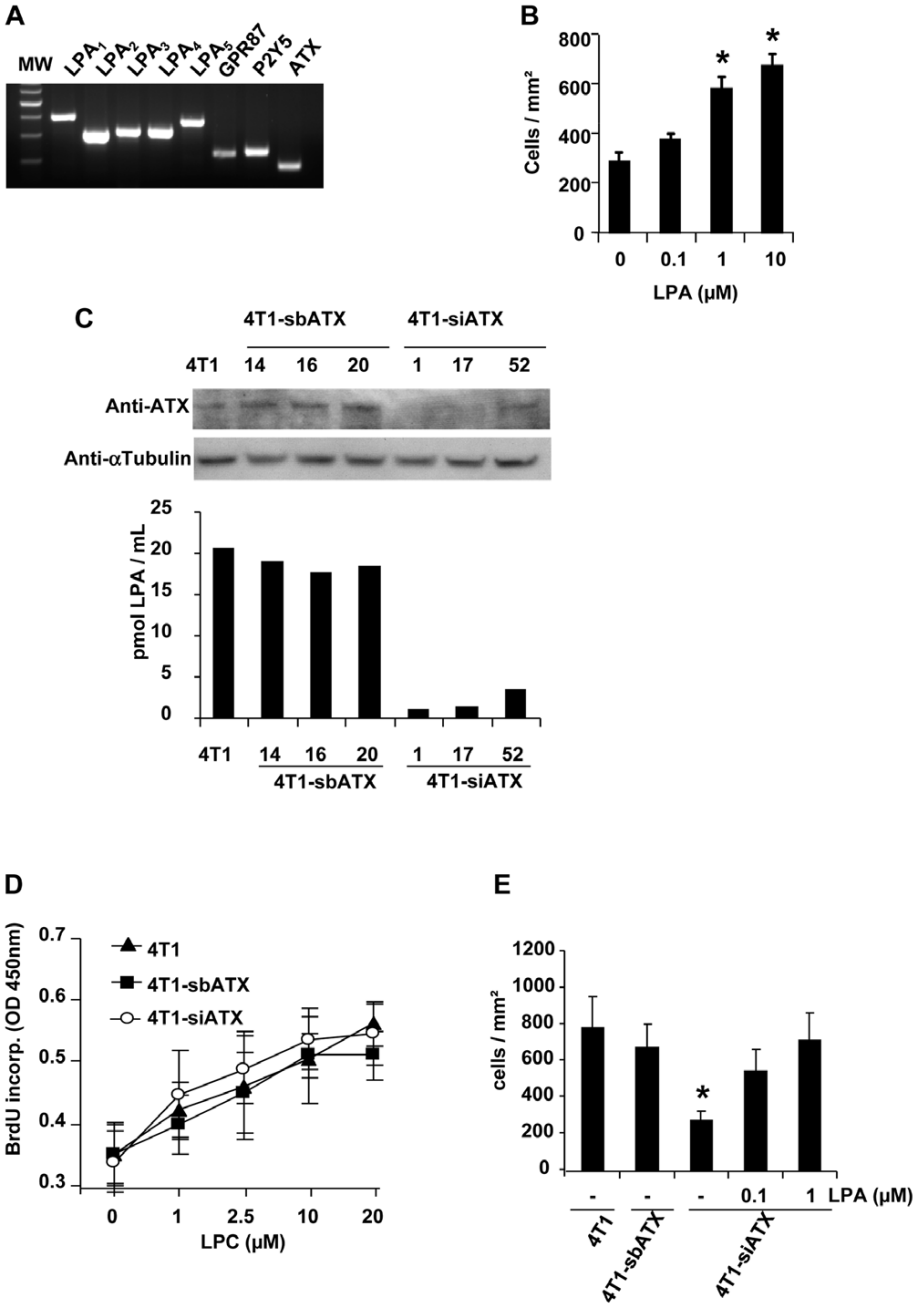
Characterization of silencing autotaxin expression in mouse breast carcinoma 4T1 cells. (A) RT-PCR amplification products for LPA receptors, LPA_1_ (1), LPA_2_ (2), LPA_3_ (3), LPA_4_ (4), LPA_5_ (5), GPR87, P2Y5, and autotaxin (ATX) from 4T1 cells total RNAs were analyzed on a 2% agarose gel. MW, molecular weight marker. (B) Cell invasion was stimulated with increased LPA concentrations used as chemoattractant. Results are the mean ± SD of cells of 3 replicates and are representative of at least 3 independent experiments. Data are expressed as the number of cells/mm^2^. (C) Autotaxin expression in 3 clones of 4T1 cells transfected with a pStrike vector coding for either irrelevant small hairpin RNAi (sbATX, clones no. 14, no. 16, no. 20) or specific small hairpin RNAi (siATX, clones no. 1, no. 17, no. 52). (Upper panel) Immunoblotting using anti-ATX polyclonal antibody or anti-?tubulin as loading control. (Lower panel) lysoPLD activity (pmol LPA/ml) measured in cell culture conditioned media. (D) Cell proliferation assessed by BrdU incorporation of 4T1 cells and a pool of three 4T1-sbATX clones (no. 14, no. 16, no. 20) or three 4T1-siATX clones (no. 1, no. 17, no. 52), in response to increased concentrations of LPC. Results are expressed in mean ± SD of 6 replicates and are representative of 3 separates experiments. (E) Invasion assay. Cells were placed in presence or absence of LPA (0.1–1 µM) in the upper chamber and FBS, used as chemoattractant, was placed in the lower chamber. Results are the mean ± SD of 3 replicates and are representative of at least 3 independent experiments. Data are expressed as the number of cells/mm^2^. *, *P*<0.05.

To address the role of autotaxin in bone metastasis formation in an immunocompetent context, we exploited the 4T1 cell line that is derived from a single mouse mammary tumor and recapitulates the distinct steps of metastasis when engrafted into the mammary gland of syngenic female BALB/C mice [29]. Firstly, we found that 4T1 cells expressed the mRNA of all forms of LPA receptors and responded to LPA stimulation (Figure 4A and 4B). Additionally, we found that 4T1 cells expressed the autotaxin transcript and protein (Figure 4A and 4C), accounting for the secretion of an enzymatically active autotaxin protein (Figure 4C). To analyze the function of endogenous expression of autotaxin by breast cancer cells in bone metastasis formation we used the RNA interference method to establish a series of three clones of 4T1 cells with stably down-regulated autotaxin expression (4T1-siATX), together with three control 4T1 clones with unaltered expression of autotaxin (4T1-sbATX) (Figure 4C). Following individual characterization of each clone for the levels of protein expressions and lysoPLD activities, subsequent *in vitro* and *in vivo* experiments were carried out using pools of the three clones for the 4T1-siATX and the 4T1-sbATX cell lines, respectively.

As previously observed for MDA-B02 cells *in vitro* (Figure 1C), LPC exhibited a mitogenic action on 4T1 cells (Figure 4D). However, silencing of autotaxin expression did not alter the proliferation of 4T1-siATX cells compared to that observed for control and parental cell lines (Figure 4D). This result was rather surprising and might be the consequence of the remaining lysoPLD activity in 4T1-siATX clones. An alternative explanation would be that the proliferation of 4T1 cells was already maximally stimulated by LPC. In contrast, using transwell migration chambers coated with Matrigel™, we observed that silencing autotaxin expression inhibited FBS-driven invasion of 4T1-siATX cells (Figure 4E). This inhibition was overcome by the addition of LPA into the cell compartment of the migration chambers demonstrating that invasion of 4T1 cells was controlled by autotaxin through its lysoPLD activity.

### Down-regulation of endogenous autotaxin expression in 4T1 cells inhibits osteolytic bone metastasis formation independently of primary tumor growth *in vivo*

**Figure 5:**
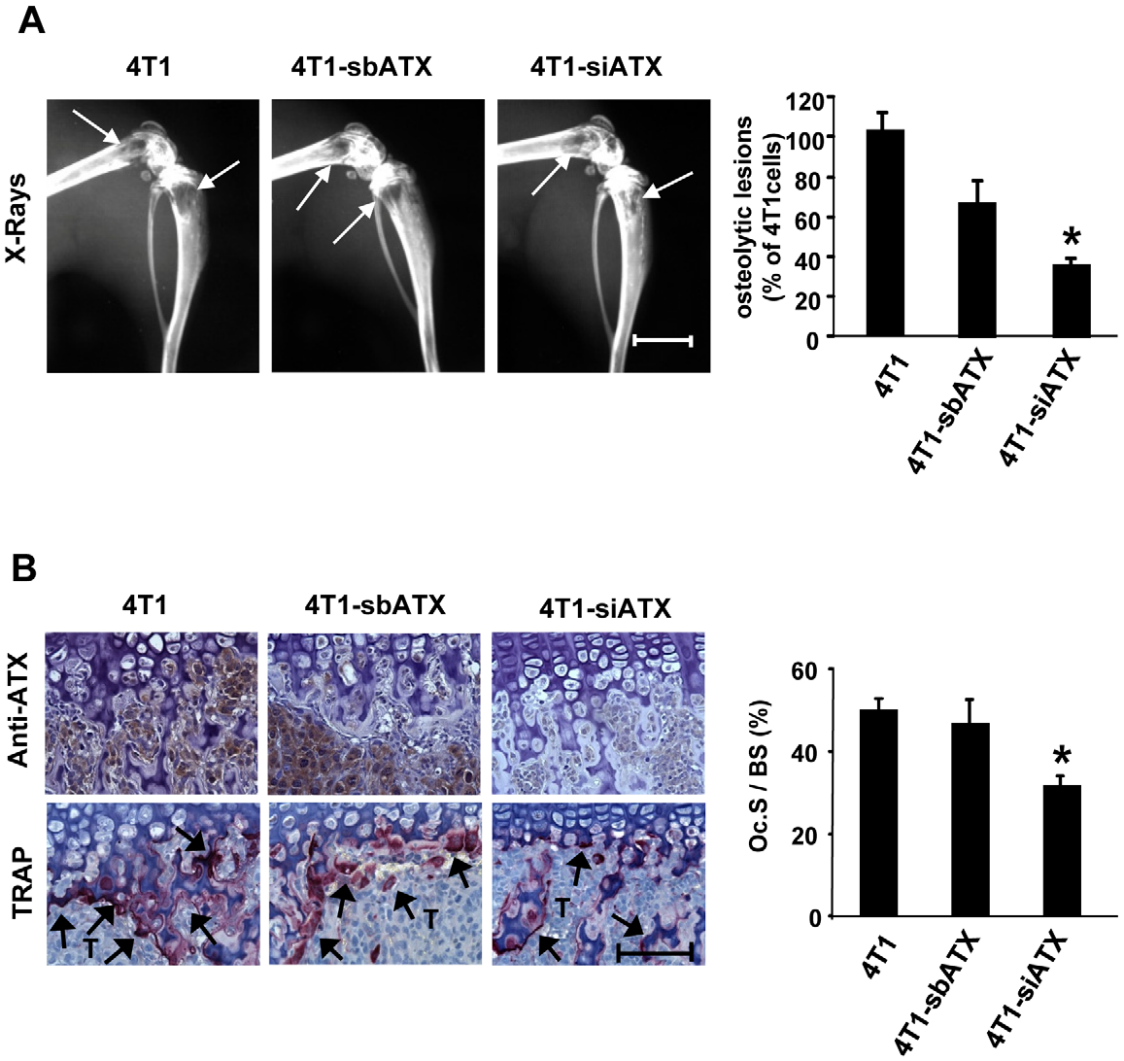
Effect of silencing autotaxin expression on osteolytic bone metastasis formation of 4T1 cells. (A) (Left panels) Representative radiographs of hind limbs from BALB/C mice 14 days after intracardiac injection of 4T1 parental cells or pools of 4T1-sbATX clones (no. 14, no. 16, no. 20) or 4T1-siATX clones (no. 1, no. 17, no. 52). Osteolytic lesions are indicated by arrows. (Right panel) Quantification of osteolytic lesion areas. Values are expressed as the % of lesion areas compared to parental 4T1 cells. Data correspond to the mean ± SE of two independent experiments of 8 to 10 animals per group. *, *P*<0.05. (B) (Left upper panels) Representative immunohistological examination of proximal tibia sections from metastatic animals 14 days after tumor cell inoculation, using the anti-ATX antibody 4F1. T indicates tumor cells. (Lower left panels) Representative histological examination of TRAP-stained proximal tibia sections from metastatic animals. T indicates tumor cells. Bone is stained in dark blue and osteoclats are stained in red (arrows). (Right panel) Quantification of active-osteoclast resorption surface per trabecular bone surface (Oc.S/BS). Results are the mean ± SE of 8-10 animals per group. *: *P*<0.05. Scale bars: 200 µm.

**Figure 6:**
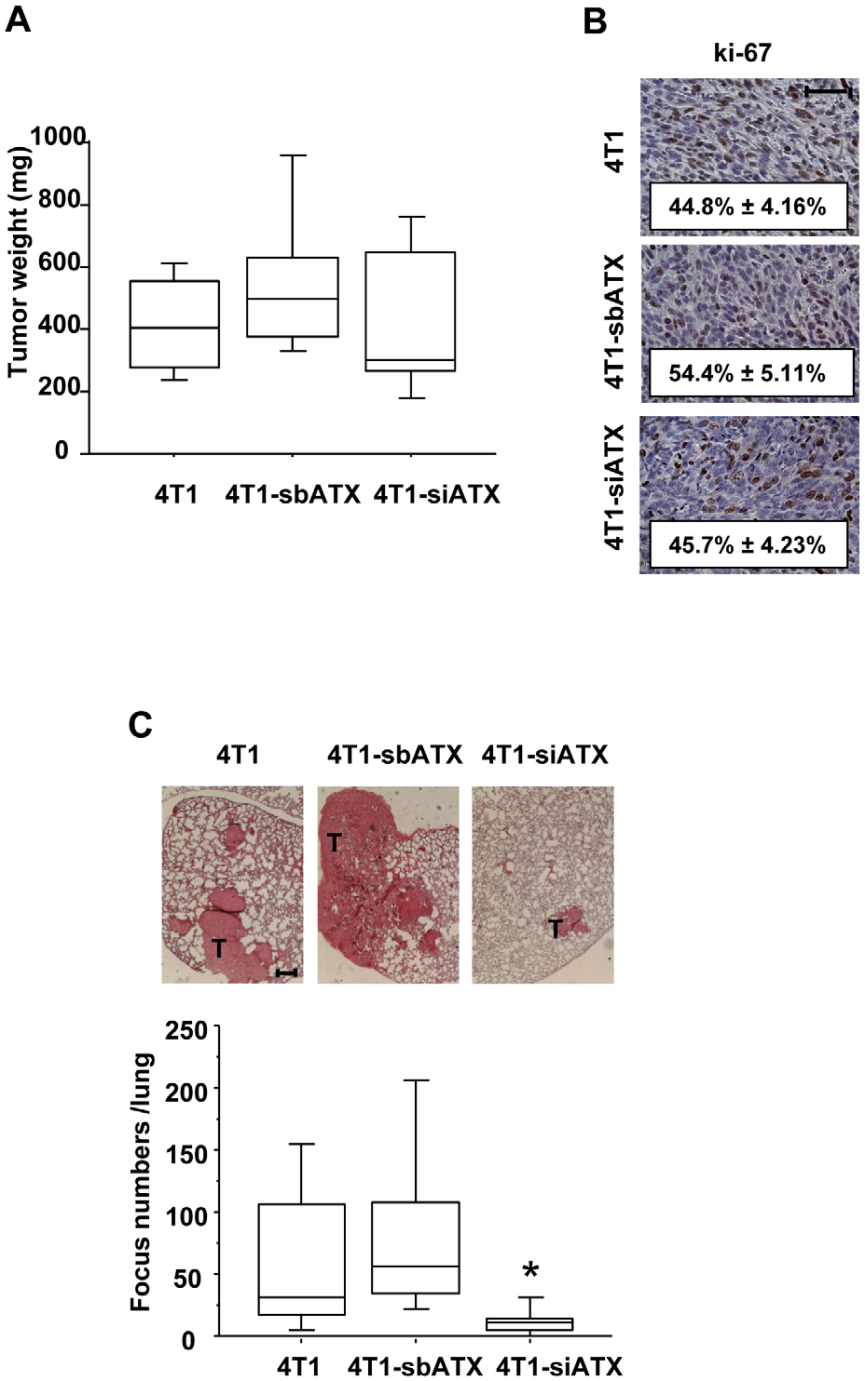
Effect of autotaxin expression in orthotopic primary tumor growth and spontaneously metastasis dissemination of mouse 4T1 cells. 4T1 parental cells, 4T1-sbATX clones and 4T1-siATX clones were injected in the mammary gland of normal syngenic female BALB/C mice. At day 14, primary tumors were resected, and weighed. (A) Box plots represent tumor weight (in mg). (B) Primary tumors were embedded in paraffin. Tumor tissue sections were analysed by mmunohistochemistry using a specific antibody directed against the nuclear ki-67 antigen. The mitotic index (numbers in each panel) was calculated as the percentage of nuclei positive for ki-67 (results are the mean ± SD, scale bar: 50 µm). (C) Animals were sacrificed 35 days after tumor cell injection and lungs were collected to quantify spontaneously metastasis formation of 4T1 cells. (Upper panels) representative photographs of lung tissue sections stained with eosin. (Lower panel) Quantification of lung metastasis foci. The number of metastatic foci was enumerated under microscope. P<0,05. T indicates metastatic foci. Scale bar: 200 µm.

To find out whether endogenous expression of autotaxin in breast cancer cells was important for bone metastasis formation, 4T1-siATX and control cell lines were injected into the left ventricle of the heart of female syngenic BALB/C mice. This strategy allows to bypass the lungs and to target the cells to other visceral organs and bone. Two weeks after cell inoculation, radiographic analyses revealed that animals bearing 4T1-siATX cells had a 50% decrease in the extent of osteolytic lesions, as compared to animals injected with 4T1-sbATX or 4T1 parental cells (Figure 5A). Histological analyses revealed that the surface of active osteoclasts per trabecular bone area located at the bone/tumor cell interface was significantly decreased in animals bearing 4T1-siATX cells, as compared to that observed in mice bearing parental or 4T1-sbATX tumor cells (Figure 5B). We have previously demonstrated that LPA is produced in the tumor microenvironment by platelets [4]. To analyze the role of endogenous autotaxin on breast cancer cells *in vivo* independently of the bone microenvironment, 4T1-siATX and control cell lines were inoculated into the fat-pad of female syngenic BALB/C mice. After two weeks, which corresponded to the same time frame of tumor growth than that used for bone metastasis experiments, primary tumors were collected. We observed that silencing autotaxin expression did not alter the growth of primary tumors since there was no significant difference in the size of 4T1-siATX tumors, as compared to those of 4T1-sbATX and parental tumors (Figure 6A). *In situ* immunodetection of the Ki-67 nuclear antigen in tumor sections did not show any difference in the proliferation of 4T1-siATX and control cells (Figure 6B). Altogether, these results indicated that endogenous expression of autotaxin controlled bone metastasis formation but not the growth of 4T1 cells *in vivo*.

In order to analyze the capacity of endogenous autotaxin to support the formation of spontaneous metastases by 4T1 cells *in vivo*, cells were injected into the mammary fat pad of female BALB/C mice. Primary tumors were grown for two weeks, at which time they were resected, allowing the emergence of spontaneous metastases. Three weeks after the resection of primary tumors, animals were sacrificed. Lungs were collected, fixed and embedded into paraffin. Lung tissue sections were examined under a microscope for the presence of metastatic foci. We found that mice inoculated with 4T1-siATX cells had a significantly lower number of lung metastases (80% reduction) as compared to mice inoculated with 4T1 parental cells (Figure 6C).

Altogether, these data indicated that the expression of endogenous autotaxin controlled the capacity of 4T1 breast cancer cells to metastasize and to induce the formation of osteolytic bone metastases independently of cell proliferation.

### Expression of autotaxin mRNA in primary tumors of patients with breast cancers

**Figure 7:**
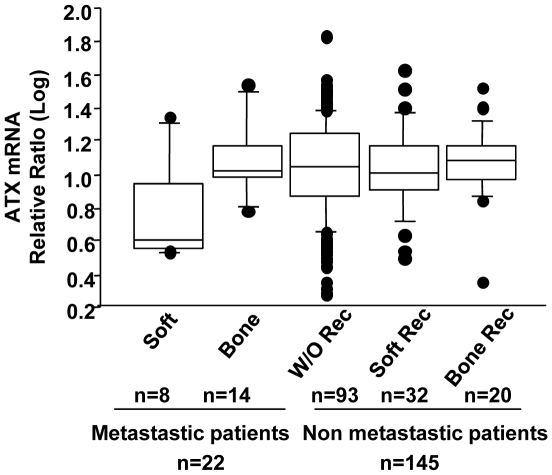
Expression of autotaxin mRNA in primary tumors of breast cancer patients. Total RNA were extracted from primary breast tumor biopsies of patients without or with metastases at the time of diagnosis. Soft and Bone represent subsets of metastatic patients with soft tissue only and bone metastases, respectively. W/O Rec represent a subset of non metastatic patients with no recurrence of metastasis during a five year period. Soft Rec and Bone Rec represent subsets of patients with recurrence of metastases to soft tissue only and to bone over a five year period, respectively. n indicates the numbers of patients in each group. Expression of Enpp2/ATX mRNA was measured by real-time quantitative by real time PCR. Quantifications were normalized to corresponding L32 RNA values. Data are given as box plots with the median. The box encompasses the 25^th^ to 75^th^ percentiles. The 5^th^ percentiles are displayed as error bars.

We then asked whether the effects of autotaxin expression observed in mouse preclinical models of breast cancers could correlate with the human disease. We analyzed the expression of the autotaxin expressing gene (*ENNP2*) by real time quantitative PCR in a series of 167 breast tumor biopsies from patients without (n = 145) or with (n = 22) identified metastases at the time of diagnosis. We observed that the expression of *ENPP2* normalized to the genes encoding ribosomal protein L32 (L32) or TATA-box binding protein (TBP, data not shown), was not significantly modified in patients with bone or soft tissue metastases at the time of diagnosis, as compared with non-metastatic patients (Figure 7). We also found similar levels of *ENPP2* mRNA in primary tumors of non-metastatic patients that had developed bone or soft tissue metastases over a five-year period (Figure 7). Then, we investigated the expression of *ENPP2* in relation to the clinical, pathological and biological characteristics, as defined in Table 1. We found that even if the absolute median values of *ENPP2* transcript levels were consistently increased together with the characteristics of poor prognostics, the differences did not reach statistically significance. This indicated that the expression of *ENPP2* in primary tumors of breast cancer patients was independent of the tumor size, grade, node, estrogen receptor (ER) and progesterone receptor (PgR) status (Table 1).

**Table 1:** Distribution of ATX mRNA in different subsets of cases defined by classical prognostic parameters in primary tumors of metastastic patients with bone metastases and of non metastatic patients without metastasis recurrence.

		Patients with bone metastases			Patients without metastasis recurrence		
		n = 14			n = 93		
		*n*	Median	p*	*n*	Median	p*
***Menopausal status***	*prepost*	*212*	10.4410.36	0.715	*6033*	10.8811.69	0.585
***Surgical tumor size***	*pT1≥ pT2*	*39*	9.8813.43		*3555*	11.3010.88	0.750
***Histological type***	*ductallobular*	*121*	10.368.15	0.285	*7713*	11.3012.19	0.823
Histological grade**	*GIGIIGIII*	*165*	6.0114.1810.08	0.248	*113920*	8.4610.8714.24	0.394
***Node status***	*pN0pN+*	*011*	10.079		*3855*	10.8711.95	0.160
***ER status***	*PositiveNegative*	*94*	9.7812.78	0.123	*8310*	11.0013.03	0.535
***PgR status***	*PositiveNegative*	*76*	9.7212.03	0.063	*7419*	10.9014.76	0.429

Data are expressed as the median of ATX/L32 mRNA ratio.

* *p* values were obtained using the non parametric Mann & Whitney test.

** in ductal carcinomas only and *p* values were obtained using the non parametric Kruskall-Wallis test.

### LPA controls directly osteoclast differentiation

**Figure 8:**
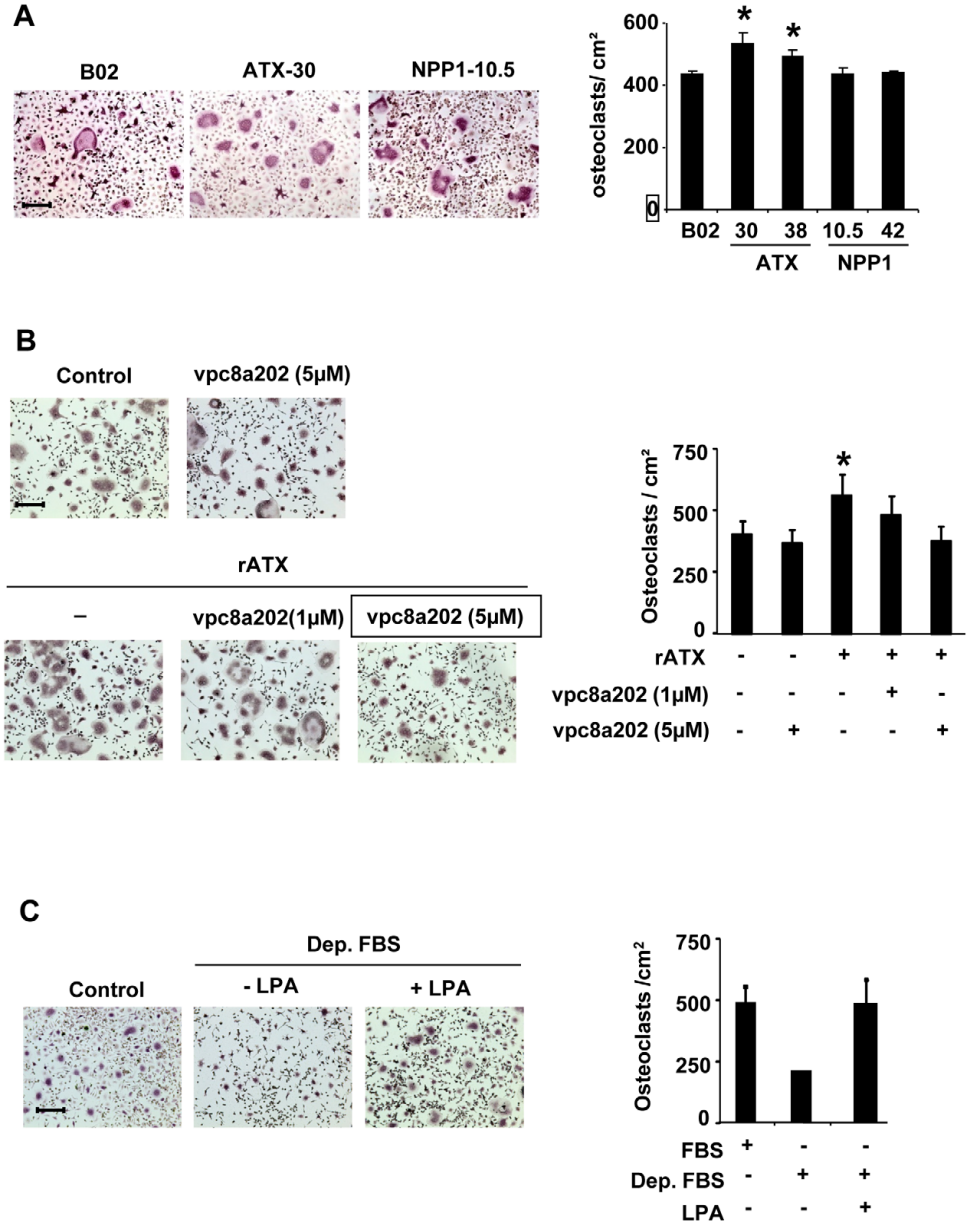
Effect of lysoPLD activity of autotaxin on osteoclastogenesis. (A) Bone marrow cells were cultured in presence of FBS (10%), mouse M-CSF, RANK-L and MDA-B02 cell or MDA-B02-ATX clone #30 or MDA-B02-NPP1 clone #10.5 conditioned media. (B) Bone marrow cells were cultured in presence of FBS (10%), mouse M-CSF, RANK-L and recombinant ATX, in absence (-) or presence of increasing concentrations of ATX inhibitor, vpc8a202. (C) Bone marrow cells were cultured in presence of mouse M-CSF, RANK-L and FBS (Control) or lipid-depleted FBS (Dep. FBS) in absence or presence of 1-Oleoyl-LPA (1 µM). (Left panels) Representative images of multinucleated cells stained for the TRAP activity. (Right panels) Quantification of osteoclast number was based on the multinucleation of TRAP-positive cells. Results are the mean ± SD of 3 separate experiments. *: *P*<0.05. Scale bars: 200 µm.

We showed previously that LPA increases the potency of tumor cells to induce the differentiation of osteoclasts *in vitro* and their recruitment *in vivo* at the bone metastatic site [4]. *In vitro*, conditioned media collected from MDA-B02-ATX clones significantly enhanced the differentiation of mature osteoclasts from bone marrow cell precursors, as compared to conditioned media prepared from parental cells or MDA-B02-NPP1 clones (Figure 8A). Moreover, recombinant autotaxin increased significantly M-CSF/RANK-L-induced osteoclast differentiation *in vitro* (Figure 8B). To determine whether the effect of autotaxin was due to its lysoPLD activity, we used a β-substituted analogue of LPA (vpc8a202) described as a specific inhibitor [30]. We observed that vpc8a202 inhibited the autotaxin-dependent increased osteoclastogenesis from bone marrow cells treated with M-CSF and RANK-L (Figure 8B). We showed previously that LPA controls indirectly breast cancer cell-induction of osteoclastogenesis through the secretion of pro-osteoclastic cytokines, IL-6 and IL-8, by tumor cells [4]. Serum is required for full osteoclast differentiation upon stimulation osteoclast precursors with M-CSF/RANK-L *in vitro* [31]. Serum contains high amounts of both LPA and LPC [32]. Therefore, our results suggested that LPA generated by autotaxin in the presence of serum might control directly osteoclast differentiation. To test this hypothesis, we treated fetal bovine serum with activated charcoal in order to remove all lipid fractions. We observed that lipid-depleted serum was not able to support osteoclastogenesis induced by M-CSF/RANK-L (Figure 8C). This inhibitory effect of lipid-depleted serum was rescued by the addition of purified LPA in the culture media (Figure 8C). This result indicated that LPA was required for serum/M-CSF/RANK-L induced osteoclastogenesis.

## Discussion

In this study we demonstrated that expression of autotaxin controlled the progression of osteolytic bone metastases induced by breast cancer cells. We showed previously that lysophosphatidic acid (LPA) produced in the tumor microenvironment controls the progression of osteolytic bone metastases of breast cancer cells and highlighted the important role of blood platelets in this process [4]. However, the molecular mechanisms involved in the production of LPA and the direct role of LPA on bone cells at the bone metastatic site are still unknown. Knockout animal analyses revealed that autotaxin controls the levels of LPA in the blood circulation [8], [9]. Forced expression of autotaxin in human breast cancer MDA-B02 cells increased the formation of osteolytic bone metastases in mice, whereas knockdown expression of endogenous autotaxin in mouse mammary tumor 4T1 cells decreased the extent of osteolytic lesions. It is known for two decades that platelets are an abundant source of LPA in the serum [33], [34]. However, Aoki's group demonstrated that LPA released directly by platelets represents only a small part of serum LPA [32]. Platelet-derived LPA is produced mainly through the action of plasma lysophospholipase D (e.g. autotaxin) on LPA precursors (LPC, lysophosphatidylethanolamine, and lysophosphatidylserine) released by activated platelets [32]. Therefore, in the context of osteolytic bone metastasis progression, the procoagulant activity of breast cancer cells is likely to induce the release of both LPA and LPA precursors upon platelet aggregation. In agreement with a local transformation of LPA precursors into LPA by tumor cell-derived autotaxin that subsequently would act as a paracine/autocrine factor, we found increased tumor burden and osteoclast-mediated bone resorption in animals bearing autotaxin-expressing tumors.

**Figure 9:**
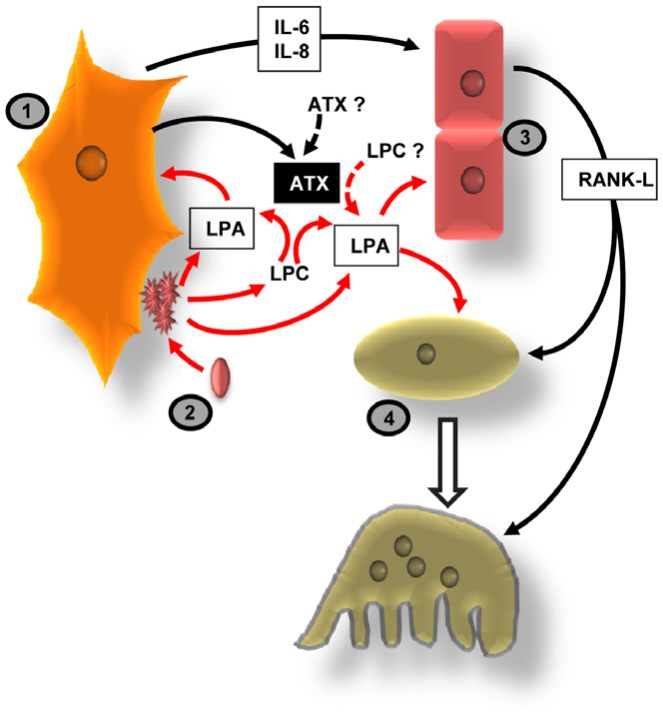
Schematic representation of LPA/autotaxin effects on the progression of osteolytic bone metastases. Bone-residing breast cancer cells (1) induce platelet (2) aggregation and the release of LPA and LPA precursors (LPC) from activated platelets. Platelet-derived LPA and LPA-derived from autotaxin (ATX) lysoPLD activity secreted by cancer cells can act on tumor cells to stimulate both tumor growth and the production of IL-6 and IL-8, which in turn induce the expression of RANK-L by osteoblasts (3) that stimulate osteoclast precursors (4) differentiation and osteoclast-mediated bone resorption. Platelet-derived LPA and LPA derived from ATX activity can act directly on osteoblasts to stimulate migration and proliferation, and on osteoclast precursors to stimulate osteoclastogenesis. Doted arrows indicate unknown origin.

Expression of *ENPP2* was detected among the set of genes that are specifically upregulated in rat bone tissue under permanent infusion with PTH(1–34) fragment [35]. Continuous exposure to PTH increases bone resorption, hypercalcemia and bone loss [36]. It is tempting to hypothesize that autotaxin would be involved in the control of osteoclast activity and bone resorption. The number of osteoclasts was specifically increased at the interface between tumors and bone in metastatic mice bearing autotaxin-expressing tumors. This could be due to an indirect action of autotaxin via an autocrine/LPA-dependant activation of tumor cells and the release of pro-osteoclastic factors [4], [5]. However, we observed that incubation of bone marrow cells *in vitro* with autotaxin-containing tumor cell conditioned media or recombinant autotaxin enhanced M-CSF/RANK-L induced differentiation of mature osteoclasts, indicating that through its lysoPLD activity autotaxin might also control directly osteoclastogenesis. Bone cells were shown to be activated by LPA [37]. LPA has a mitogenic and chemotatic activity on osteoblasts and induces dentritic outgrowth of osteocytes [38], [39], [40]. LPA was also shown to cooperate with 1alpha, 25(OH)Vitamin D to stimulate osteoblastic cell activities [41]. Here, we found that LPA present in the serum was required to form mature osteoclasts *in vitro* from bone marrow cell precursors. Therefore, in addition to a role on tumor growth, both at primary and bone metastatic sites, LPA might also control the progression of osteolytic bone metastases through a direct action on bone cells (Figure 9).

Human MDA-B02 cells used in our study do not express autotaxin at steady state, indicating that expression of autotaxin was not involved in the tropism of breast cancer cells to the bone tissue. This might explain why expression of autotaxin gene (*ENPP2*) was not identified among the set of genes that defined the gene signature of bone-seeking breast cancer cells [42], [43]. However, expression of autotaxin both in human and mouse breast cancer cells controlled the progression of osteolytic lesions, once located in the bone tissue. Bone metastasis is the site of complex cross-interactions occurring between tumor cells and the bone microenvironment. Genes encoding for parathyroid hormone related protein (PTHrP), cyclo-oxygenase 2 (Cox2) and osteomimetic factors by tumor cells do not belong to the gene signature of bone-seeking breast cancer cells, but are known to play active roles during the progression of bone metastases [44], [45], [46]. Our results strongly suggest that autotaxin belongs to these additional factors that control the expansion of osteolytic bone metastases in breast cancers.

Taghavi and collaborators observed that LPA receptors are conditionally tumorigenic in mice [47]. A recent study showed that transgenic overexpression of autotaxin or LPA1-3 receptors in the mouse mammary gland is sufficient to initiate breast cancers in mice, demonstrating that activation of the autotaxin/LPA track induces carcinogenesis [25]. This confirms previous reports indicating that expression of autotaxin is increased in tumors compared to normal tissues of origin [26]. However, the role of the autotaxin/LPA axis during the progression of breast cancers in human is not well characterized yet. Increased expression of LPA_2_ and LPA_3_ receptors correlates with late stages of ovarian cancers, whereas expression of LPA_1_ receptor links to the inhibition of the progression of ovarian tumors. Specific variation of LPA receptor expression was not found in DNA microarray databases of different stages and grades of breast cancers, suggesting that LPA receptors are more prone to control primary tumor progression of ovarian tumors than breast cancers [48]. We found that the autotaxin transcript levels in primary tumors of breast cancer patients was an independent factor from the tumor size, grade, metastasis, node, ER and PgR status. This suggested that autotaxin expression would not be predictive for both the progression and metastatic dissemination of breast cancer tumors.

In conclusion, in addition to the predominant role of autotaxin/LPA track in the carcinogenesis of breast cancers our results demonstrated that activation of autotaxin/LPA axis in breast cancer cells controlled the progression of osteolytic bone metastases by stimulating directly both cancer cells and osteoclasts. Osteophylic cancers such as breast, thyroid, prostate and lung cancers, share many common mechanisms during the progression of bone metastases [2]. In addition, expressions of autotaxin and LPA receptors are detected in these types of cancers [21], [23], [49]. Altogether, our results suggested that targeting autotaxin/LPA track might contribute to the development of new therapies to improve the care of patients with osteophylic solid tumors.

## Materials and Methods

### Ethics statement

The mice used in our study were handled according to the rules of Décret N° 87–848 du 19/10/1987, Paris. The experimental protocol have been reviewed and approved by the Institutional Animal Care and Use Committee of the Université Claude Bernard Lyon-1 (Lyon, France). Studies were routinely inspected by the attending veterinarian to ensure continued compliance with the proposed protocols. BALB/C and BALB/C nude mice, 4 weeks of age, were housed under barrier conditions in laminar flow isolated hoods. Autoclaved water and mouse chow were provided ad libitum. Animals bearing tumor xenografts were carefully monitored for established signs of distress and discomfort and were humanely euthanized when these were confirmed. Studies involving human primary breast tumors were performed according to the principles embodied in the Declaration of Helsinki. Tissue biopsies were obtained as part of surgical treatments for the hormone receptor content determination. Remaining samples were included anonymously in this study. All human experiments were approved by the Experimental Review Board from the Laennec School of Medicine that waived the need for consent.

### Drugs and reagents

Lysophosphatidic acid (LPA, Oleoyl C18∶1) and lysophosphatidylcholine (LPC) were obtained from Avanti Polar Lipids. Activated charcoal was obtained from VWR international. The competitive inhibitor of LPA signaling pathways dependent on LPA_1_ and LPA_3_ receptors, Ki16425, was obtained from DebiopharmGroup. Vpc8a202 was kindly provided by Pr. K.R. Lynch (University of Virginia, Charlottesville,VA)[30]. Recombinant Human autotaxin was purchased from R&D System.

### Cell lines and transfection

Cell lines (MDA-B02 and 4T1) and transfectants were cultured in complete media, DMEM medium (Invitrogen), 10% (v/v) fetal bovine serum (FBS, Perbio) and 1% penicillin/streptomycin (Invitrogen), at 37°C in a 5% CO_2_ incubator. Human MDA-MB-231 and mouse 4T1 breast cancer cell lines were obtained from the American Type Culture Collection. Characteristics of MDA-MB-231/B02 (MDA-B02) breast cancer cells were described elsewhere [50]. The cDNA encoding the rat autotaxin and mice NPP1 were amplified by PCR using the plasmid HA-RnNPP2-Myc and HA-NPP1-Myc as a template and 2 oligonucleotide primers (5′-GCAGAGCTGGTTTAGTGAAC-3′ and 5′-CCTCTACAAATGTGGTATATGGC-3′). The bidirectional vectors pBiL/ATX and pBiL/NPP1 were constructed by inserting into the pBiL plasmid (Clonetech) the NotI/NheI PCR fragment encoding the autotaxin and NPP1 sequence, respectively. MDA-MB-231/B02-tet-Off cells were cotransfected with pBiL/ATX or pBiL/NPP1 together with a vector conferring puromycin resistance (pPur; Clontech). Selection of the clones was obtained after growing the cells for 2 weeks in the presence of puromycin (2 µg/mL). Luciferase induction was used to select clones among stable transfectants. Two autotaxin transfectants (clones no. 30 and 38) and two NPP1 transfectants (clones no. 10.5 and 41) were used in the present study.

We designed small hairpin RNAs (SiRNA) and corresponding SbRNA sequences directed to autotaxin mRNA target sites based on the mouse sequence (GenBank accession NM_015744) using the SIRNA TARGET DESIGNER (Promega). Pairwise oligonucleotides for mouse siRNA-ATX 5′-ACCGCCATCGGCGTCAATCTCTAAGTTCTCTAGAGATTGACGCCGATGGCTTTTTCC-3′, 5′-TGCAGAAAAAGCCATCGGCGTCAATCTCTAGAGAACTTAGAGATTGATTGACGCCGATGG-3′ (target nucleotide site: 97–115) as well as control oligonucleotides with scrambled sequences (sbl-ATX) were cloned into psiSTRIKE puromycin vector containing the U6 promoter (Promega). Plasmids were transfected into 4T1 cells using the Transfast reagent (Promega). Cells were cultured for 2 weeks in presence of puromycin (2 µg/mL) and 4T1-siATX and 4T1-sbl clones were isolated using cloning cylinders. Autotaxin expression was assessed by western-blotting and measure of lyso-PLD activity. Three 4T1SiRNA-ATX stable transfectants (clones no. 1, 17 and 52) and three 4T1SblRNA-ATX stable transfectants (clones no. 14, 16 and 20) were used in the present study.

### Reverse transcription and polymerase chain reaction (RT-PCR)

Total RNA from 4T1 cells were extracted using Nucleospin RNAII kit (Macherey-Nagel) and cDNA were synthesized using iScript cDNA Synthesis kit (Biorad). PCR reactions were run using SYBR® Green qPCR kit and sets of specific primers, LPA1-F (5′-ATCTTTGGCTATGTTCGCCA-3′) and LPA1-R (5′-TTGCTGTGAACTCCAGCCA-3′) for LPA_1_; LPA2-F (5′-GTCAAGACGGTTGTCATCATTCT-3′) and LPA2-R (5′-GAAGCATGATCCGCGTGCT-3′) for LPA_2_; LPA3-F (5′-GAAGCATGATCCGCGTGCT-3′) and LPA3-R (5′-TCATGATGGACATGTGTGTTTGC-3′) for LPA_3_; LPA4-F (5′- GCATTGTTGACATTAGTGGTGGA -3′) and LPA4-R (5′- AACCTGGCCCTCTCTGATTT-3′) for LPA_4_; LPA5-F (5′-CCGTACATGTTCATCTGGAAGAT-3′) and LPA5-R (5′-CAGACTAATTTCTCTTCCCACCT-3′) for LPA_5_; GPR87-F (5′-CAGACTAATTTCTCTTCCCACCT-3′) and GPR87-R (5′-GGGGATTCTGCACAAGTGAT-3′) for GPR87; P2Y5-F (5′-GAGCAGTCCCAGTGGCTTAG-3′) and P2Y5-R (5′-TGTTTCCAACTGCTGCTTTG-3′) for P2Y5; ATX-F (5′-GCCCTGATGTCCGTGTATCT-3′) and ATX-R (5′-CGTTTGAAGGCAGGGTACAT-3′) for autotaxin. Complementary DNAs were amplified by PCR for 35 cycles and PCR products were analyzed by electrophoresis on a 2% agarose gel stained with ethidium bromide. Expression of Enpp2/ATX mRNA was quantified by real-time quantitative RT-PCR on an Eppendorf Mastercycler® RealPlex (Invitrogen) using the SYBR® Green PCR kit (Finnzymes). Quantifications were normalized to corresponding RNA L32 and TBP values. The cDNAs were amplified by PCR for 35 cycles with the following specific PCR primers: human ATX, 5′- ACAACGAGGAGAGCTGCAAT-3′ (forward) and 5′- AGAAGTCCAGGCTGGTGAGA-3′ (reverse); Human L32, 5′- CAAGGAGCTGGAAGTGCTGC-3′ (forward) and 5′- CAGCTCTTTCCACGATGGC-3′ (reverse); Human TBP, 5′-TGGTGTGCACAGGAGCAAG-3′ (forward) and 5′-TTCACATCACAGCTCCCCAC-3′ (reverse). Each cycle consisted of 10 s of denaturation at 95°C, 15 s of annealing at 67°C, and 10 s of extension at 72°C.

### Quantification of lysophosphilipase D (lysoPLD) and phosphodiesterase (PDE) activities

lysoPLD activity was measured by conversion of radiolabeled LPC into radiolabeled LPA as described previously [51]. Briefly, a solution of [^14^C]palmitoyl-lysophosphatidylcholine at 0.0025 µCi/µL in DMEM supplemented with 1% free acid BSA was first prepared, and 20 µl of this solution was incubated with 500 µl of thawed CM plus 1 µl of sodium orthovanadate for 90 min at 37°C. At the end of the incubation period, phospholipids were extracted with 500 µl of 1-butanol, evaporated, spotted on a silica gel 60 TLC glass plate, and separated using CHCl_3_/MeOH/NH_4_OH (60∶35∶8) as the migration solvent. The plate was autoradiographed overnight at −80°C using Biomax-MS film (Kodak) to localize radiolabeled LPA spots, which were scraped and counted with 3 mL of scintillation mixture. Nucleotide phosphodiesterase activities were determined from the release of *p*-nitrophenolate from *p*-nitrophenyl thymidine 5′-monophosphate. Briefly, the samples were incubated at 37°C with 5 mM of the substrate, 5 mM CaCl_2_, 5 mM MgCl_2_ and 100 mM Tris/HCl at pH 9.0 in a total volume of 35 µL. The reaction was stopped by the addition of 200 µL of 3% (v/v) trichloroacetic acid. Subsequently, the mixture was neutralized with NaOH and *p*-nitrophenolate was quantified colorimetrically at 405 nm.

### Western-blot analysis

Cells were cultured in 5 ml DMEM without FBS for one day. Conditioned culture media were concentrated to 60 µl using Centicon-Y30 (Millipore). Cell lysates were prepared from cells cultured in complete medium. Protein were electrophoresed on a 7% SDS polyacrylamide gel and transferred onto an Immobilon transfer membrane (Millipore). Membranes were incubated with 5% low fat-milk and 0.1% Triton X-100, pH 7.4 in PBS for one hour at room temperature followed by an overnight incubation with anti-ATX antibody (Cayman) or with anti-α-tubulin antibody (Sigma Aldrich). Autotaxin and α-tubulin were visualized using horseradish-peroxidase-donkey anti-rabbit IgG or anti-mouse IgG (Amersham) and enhanced chemiluminenscence (Amersham). For detection of HA-NPP1-myc, non-specific binding sites were blocked with 3% bovine serum albumin (Serva) and 0.1% Tween-20 in PBS. Following overnight incubation with anti-Myc (clone 9E10) monoclonal anibodies, HANPP1myc was visualized using horseradish-peroxidase-goat anti-mouse IgG (Dako) and enhanced chemiluminesence (Perkin Elmer).

### Cell proliferation assay

Experiments were carried out in conditions described previously [23]. Briefly, MDA-B02 cells (8×10^3^) and 4T1 cells (2.5×10^3^) were seeded in 48-well and 96-well plates, respectively, and cultured in complete medium for 24 h. Then, cells were synchronized in the presence of serum-free medium for 24 h. Then, cell proliferation was evaluated following BrdU incorporation for 7 h and the use of the cell proliferation ELISA kit (Roche).

### Cell invasion assay

Invasion assays were carried out using Bio-Coat migration chambers (Becton Dickinson) with 8 µm filters previously coated with Matrigel as described previously [52]. MDA-B02 cells (5×10^4^) or 4T1 cells (2.10^5^) were plated in the upper chambers and and the chemoattractant (10% FBS) in the lower chambers. After incubation for 24 h at 37°C in 5% CO_2_ incubator, cells that had migrated through the filters were fixed and stained. The membranes were mounted on glass slides, and cells from 10 random microscopic fields (x400 magnification) were counted. All experiments were run in duplicate, and invasion was expressed in terms of cells/mm^2^.

### Patients and tumor characteristics

Patients were selected according to the following criteria: primary breast tumor without inflammatory feature, no previous treatment. Patients tumors were provide by three medical centers (Centre Hospitalier Régional Annecy, Chirurgie Oncologique Centre Hospitalier Universitaire Lyon-Sud, and Clinique Mutualiste Saint Etienne, France) in which patients were included between October 1994 and October 2001. Two groups of patients were defined according to identified metastases at the time of diagnosis (n = 22) or no identified metastases (n = 145). Breast cancer tissue biopsies were obtained by surgery, selected by the pathologist and immediately stored in liquid nitrogen until processing. The biopsies were pulverized using a “Mikro-Dismembrator” (B. Braun Biotech International, Melsungen, Germany) and total RNAs were extracted using TRI Reagent (Sigma, St Louis, MO). To remove any genomic DNA contamination, total RNAs were treated with RNAse-free DNAse I and purified using RNeasy micro columns (Qiagen, Hilden, Germany). RNAs quality was verified using an Agilent Bioanalyser 2100 (Agilent Technologies, Santa Clara, CA).

### Animal studies

Bone metastasis experiments using MDA-B02 cells and transfectants were performed in female BALB/C *nude* mice of 4 weeks of age as previously described [4]. Cells were suspended at a density of 5×10^5^ cells in 100 µl of PBS and inoculated intravenously into animals. Bone metastasis experiments using 4T1 cells and transfectants were performed using female BALB/cByJ mice of 6 weeks of age. Cells were suspended at a density of 10^5^ cells in 100 µl of PBS and administered to the animals by intracardiac injection. Radiographs (LifeRay HM Plus, Ferrania) of animals were taken at day 28 and 14 after inoculation with MDA-B02 cells and 4T1cells, respectively, using a cabinet X-ray system (MX-20; Faxitron X-ray Corporation). Then, animals were sacrificed by cervical dislocation and metastatic hind limbs were collected for histology and histomorphometric analyzes. Areas of osteolytic lesions were measured using the computerized image analysis system MorphoExpert (Exploranova). The extent of bone destruction for each animal was expressed in mm^2^.

Tumor fad pad experiments were performed using 4T1 parental cells or pools of 3 independent clones of 4T1-siRNA-ATX or 4T1-sblRNA-ATX cells (10^5^ in 10 µl of PBS) injected into the fat pad of the 4^th^ mammary gland of female BALB/C mice of 6 weeks of age (Charles River). Tumor weights were determined 14 days after inoculation. For spontaneous metastasis dissemination studies 14 days after tumor cell injection, animals were anesthetized and primary tumors were surgically removed. Mice were then followed for an additional 3-week observation at which time they were sacrificed and lungs collected for histological analysis.

### Bone histomorphometry and histology

Hind limbs from animals were fixed, decalcified with 16% EDTA and embedded in paraffin. Five-µm tissue sections were stained with Goldner's Trichrome and proceeded for histomorphometric analyzes to calculate the BV/TV ratio (percentage of bone tissue) and the TumV/TV ratio (percentage of tumor tissue). The *in situ* detection of osteoclasts was carried out on metastatic bone tissue sections using the tartrate-resistant acid phosphatase (TRAP) activity kit assay (Sigma). The resorption surface (Oc.S/BS) was calculated as the ratio of TRAP-positive trabecular bone surface (Os.S) to the total trabecular bone surface (BS) using the computerized image analysis system MorphoExpert (Exploranova).

### Immunohistochemistry

Resected primary tumors and metastatic hind limbs were fixed and embedded in paraffin. Five µm sections were subjected to immunohistochemistry using a rat anti-mouse Ki67 monoclonal antibody that specifically recognizes proliferative cells (DakoCytomation) and a rat anti-ATX monoclonal antibody (clone 4F1). Sections were de-paraffinized in methylcyclohexan, hydrated through a graded series of ethanol, then immersed in a peroxidase blocking reagent (DakoCytomation) 5 min. Sections were incubated with normal goat serum for 30 min and incubated overnight at 4°C in humid chambers with primary antibody to Ki67 (dilution1∶25) and to ATX (dilution 1∶200). The slides were incubated with biotinylated polyclonal rabbit anti-rat immunoglobulin (DakoCytomation) for 45 min. After washing, the slides were treated with peroxidase conjugated streptavidin (DakoCytomation) for 45 min and developed by addition of a solution of 3,3′-diaminobenzidine tetrahydrochloride (DakoCytomation). Light counterstaining was performed with Mayer's hematoxylin. The number of nuclei immunostained for Ki-67 was counted under a microscope. The mitotic index was calculated as the ratio of the number of Ki-67 positive nuclei to the total nucleus number per field and expressed as the percentage of Ki-67-positive nuclei.

### Osteoclastogenesis assay

Bone marrow cells from hind limbs of OF1 male mice were collected and seeded in 12-well tissue culture plates at a density of 2×10^5^ cells per well in α-MEM medium (Invitrogen) supplemented with macrophage–CSF (R&D Systems), receptor-activated nuclear receptor factor κB ligand (RANK-L). Culture media were then supplemented with 10% FBS, lipid-depleted FBS or conditioned media collected from tumor cells, in presence or absence of 1-oleoyl LPA, recombinant autotaxin or vpc8a202. After 6 days, mature osteoclasts were enumerated under a microscope on the basis of the number of nuclei (more than three nuclei) and the tartrate-resistant acid phosphatase (TRAP) activity (Sigma). Results were expressed as the number of osteoclasts per cm^2^.

### Statistical analysis

Data were analyzed with the Stat-View 5.0 software using unpaired Student's *t* test for *in vitro* and *in vivo* studies, and the non parametric Mann Whitney test or Kruskall-Wallis test for the clinical study. *P* values less than 0.05 were considered statistically significant.
